# Alcohol Co-Administration Changes Mephedrone-Induced Alterations of Neuronal Activity

**DOI:** 10.3389/fphar.2021.679759

**Published:** 2021-04-28

**Authors:** Milo Grotell, Bjørnar den Hollander, Aaro Jalkanen, Essi Törrönen, Jouni Ihalainen, Elena de Miguel, Mateusz Dudek, Mikko I. Kettunen, Petri Hyytiä, Markus M. Forsberg, Esko Kankuri, Esa R. Korpi

**Affiliations:** ^1^ Department of Pharmacology, Faculty of Medicine, University of Helsinki, Helsinki, Finland; ^2^ School of Pharmacy, Faculty of Health Sciences, University of Eastern Finland, Kuopio, Finland; ^3^ Kuopio Biomedical Imaging Unit, A.I. Virtanen Institute for Molecular Sciences, Faculty of Health Sciences, University of Eastern Finland, Kuopio, Finland

**Keywords:** stimulant, ethanol, co-consumption, MEMRI, golgi-staining, methamphetamine, mephedrone

## Abstract

Mephedrone (4-MMC), despite its illegal status, is still a widely used psychoactive substance. Its effects closely mimic those of the classical stimulant drug methamphetamine (METH). Recent research suggests that unlike METH, 4-MMC is not neurotoxic on its own. However, the neurotoxic effects of 4-MMC may be precipitated under certain circumstances, such as administration at high ambient temperatures. Common use of 4-MMC in conjunction with alcohol raises the question whether this co-consumption could also precipitate neurotoxicity. A total of six groups of adolescent rats were treated twice daily for four consecutive days with vehicle, METH (5 mg/kg) or 4-MMC (30 mg/kg), with or without ethanol (1.5 g/kg). To investigate persistent delayed effects of the administrations at two weeks after the final treatments, manganese-enhanced magnetic resonance imaging brain scans were performed. Following the scans, brains were collected for Golgi staining and spine analysis. 4-MMC alone had only subtle effects on neuronal activity. When administered with ethanol, it produced a widespread pattern of deactivation, similar to what was seen with METH-treated rats. These effects were most profound in brain regions which are known to have high dopamine and serotonin activities including hippocampus, nucleus accumbens and caudate-putamen. In the regions showing the strongest activation changes, no morphological changes were observed in spine analysis. By itself 4-MMC showed few long-term effects. However, when co-administered with ethanol, the apparent functional adaptations were profound and comparable to those of neurotoxic METH.

## Introduction

During the last couple of decades, there has been an increasing supply of substituted cathinones in the illegal drug market (Vardakou et al., 2011; Deluca et al., 2012; EMCDDA, 2018). Generally, these substances initially gain popularity as legal alternatives to existing amphetamine-type stimulants. As their popularity grows, they are subsequently banned by national governments and once banned, they become available on the illegal drug market next to already controlled substances. One of the better-known substituted cathinones is mephedrone (4-methylmethcathinone, 4-MMC), the usage of which has remained high even after its classification as illegal (Deluca et al., 2012; Papaseit et al., 2020).

4-MMC shows many pharmacological similarities with methamphetamine (METH), with both substances producing rapid and substantial increases in accumbal extracellular dopamine (DA) and serotonin levels *in vivo* (Kehr et al., 2011). The effects of both METH and 4-MMC are mainly mediated via increased release and blockade of reuptake of DA (Cho and Melega, 2002; Winstock et al., 2011b; Hadlock et al., 2011; Lopez-Arnau et al., 2012; Martinez-Clemente et al., 2012; Korpi et al., 2015). However, long-term neurotoxic effects of mephedrone and METH appear to differ substantially. While METH is known to produce substantial and long-lasting reductions in monoamine levels and other markers of DA neurotoxicity, such findings are generally not replicated with 4-MMC. In fact, several studies have found little evidence of mephedrone neurotoxicity when administered under normal conditions (Angoa-Perez et al., 2012; Baumann et al., 2012; Angoa-Perez et al., 2013; Angoa-Perez et al., 2014; den Hollander et al., 2015; Ciudad-Roberts et al., 2016). Nonetheless, 4-MMC neurotoxicity can be precipitated when the drug is administered under circumstances known to exacerbate stimulant neurotoxicity, such as high ambient temperatures (Hadlock et al., 2011; Lopez-Arnau et al., 2015; Pantano et al., 2017).

In recreational settings, stimulants such as 4-MMC and METH are often used in conjunction with alcohol (ethanol, EtOH) (Winstock et al., 2011a; Papaseit et al., 2020). Alcohol use is a known exacerbating factor for stimulant neurotoxicity and has been shown to increase or precipitate neurotoxic effects of MDMA and METH (Izco et al., 2007; Rodriguez-Arias et al., 2011; Blaker and Yamamoto, 2018; Blaker et al., 2019). This raises the question whether alcohol could also precipitate 4-MMC neurotoxicity. Currently, very little is known about this. One study reported evidence of neurotoxicity when 4-MMC was co-administered with EtOH, but this study also administered the drug at high ambient temperatures, making it difficult to conclusively attribute the effects to EtOH (Ciudad-Roberts et al., 2016). Another study in mice reported EtOH increased 4-MMC-induced conditioned place preference but did not assess toxicity (Ciudad-Roberts et al., 2015). Interestingly, the acute effects of 4-MMC alone or in combination with EtOH was investigated in a clinical study which demonstrated that EtOH increased the cardiovascular effects of the drug as well as self-reported euphoria. However, this study did not investigate long-term neurocognitive effects (Papaseit et al., 2020). Further investigating this question is of importance considering that 4-MMC is often consumed together with EtOH and will help inform both drug users and healthcare providers of potential risks and help guide the development of an evidence-based harm reduction approach.

We previously used manganese-enhanced magnetic resonance imaging (MEMRI) to assess the long-term effects of 4-MMC and METH in rats and showed that METH produced a pattern of widespread neuronal deactivation in monoamine-rich brain areas 2 weeks following a binge-dosing regimen; 4-MMC, conversely, produced an effect that was limited primarily to the parietal cortex, hypothalamus and hippocampus and was characterized by neuronal activation rather than deactivation (den Hollander et al., 2015). MEMRI signal suppression has previously been associated with neurotoxicity in a study where MPTP-induced loss of DA neurons in substantia nigra produced decreased neuronal activation by MEMRI (Weng et al., 2016). The MEMRI method is based on the fact that manganese functions as a Ca^2+^ analogue *in vivo*; manganese influx into neuronal cells via the Ca^2+^ channels during fast neuronal depolarization and subsequent distribution throughout the neuron and axons thereby represent a measure of mean neuronal activity over time (Bedenk et al., 2018; Svehla et al., 2018).

Here, we employed MEMRI to investigate the long-term effects of EtOH co-administration with 4-MMC or METH on neuronal activity. Additionally, we used Golgi staining to evaluate potential changes in neuronal morphology in regions of interest identified based on MEMRI neuronal activity patterns. We show that EtOH modifies the long-term effects of 4-MMC on neuronal activity, resulting in a similar pattern of deactivation as observed after METH administration.

## Materials and Methods

### Animals

A total of 48 juvenile male Wistar rats (RccHan:WIST, supplied by the Laboratory Animal Center, University of Eastern Finland, Kuopio, Finland; 8 weeks old at the start of the experiment) were used in this study. The rats were randomly allocated to six treatment groups, with eight rats per group. The rats were housed in one-animal, open-air cages containing woodchip bedding and environmental enrichment including hardwood blocks and plastic shelter tubes, with food pellets (Teklad 2016S, Envigo, Netherlands) and tap water available *ad libitum*. The rats were maintained under a 12-h light–dark cycle with lights on from 7.00 a.m. All treatments were given, and all tests were performed during daytime (07:00 a.m.–07:00 p.m.). All animal experiments were performed in accordance with European Union guidelines (Directive 2010/63/EU and the guideline 2007/526/EC) and approved by the Division of Health and Social Services, Legality and Licensing of the Regional State Administrative Agency for Southern Finland (license number ESAVI-2016–001158). All effort was taken to minimize animal suffering and the number of animals used.

### Drugs

METH was purchased from Sigma-Aldrich (St. Louis, MO, United States) and ethanol (EtOH 99.96%) from VWR (Pennsylvania, United States); 4-MMC was synthesized in-house (Siivonen et al., 2018). Other products were acquired from Sigma-Aldrich unless specified otherwise. All drugs were dissolved in saline on the day before use and administered intraperitoneally at a volume of 1 ml/kg (METH and 4-MMC) or 10 ml/kg (EtOH) as two separate injections at approximately 8-h intervals.

### Dose Selection

**FIGURE 1:**
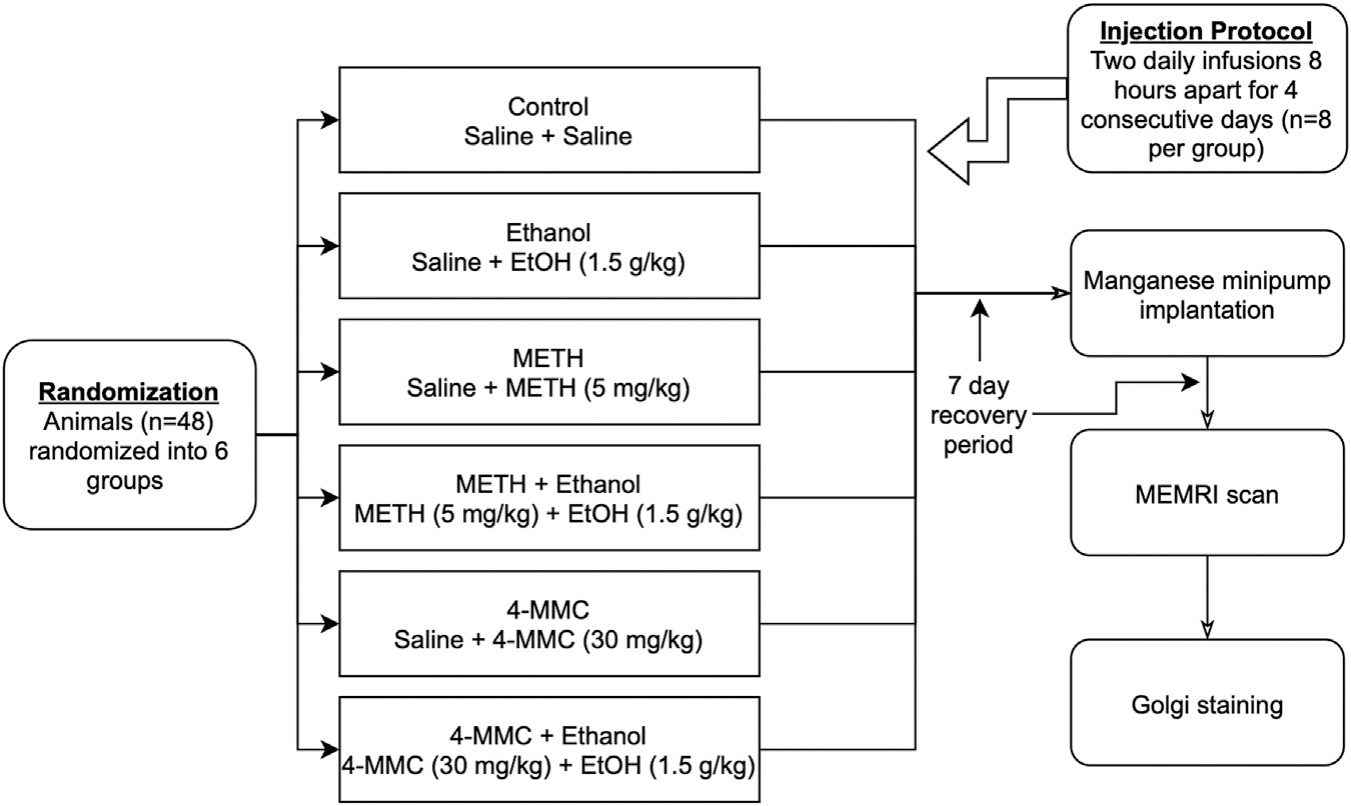
Experimental design shown as a flowchart. 4-MMC, 4-methylmethcathinone; EtOH, ethanol; METH, methamphetamine.

The 5 mg/kg METH and 30 mg/kg 4-MMC doses employed in this study were in line with those used previously and based on an estimation of the relative difference in potency between 4-MMC and METH (den Hollander et al., 2015). The 1.5 g/kg EtOH dose was chosen to produce a blood alcohol level of approximately 0.2% (by vol; 200 mg/dl, 43.3 mmol/L) that is characteristic for hazardous binge drinking (Vonghia et al., 2008). The aim of the dosing regimen was to mimic a pattern of heavy, recreational binge-abuse. As such, all treatments were administered twice daily (morning and afternoon, approximately 8 h apart) for four consecutive days. An overview of drug and EtOH treatments administered to the various experimental groups is shown in Figure 1.

### Overview of Experimental Design

After a habituation period of four days in groups of four animals, the experiment began on Day 1 with the 4-days drug binge treatment regimen of METH and 4-MMC alone or in combination with EtOH, with additional saline and EtOH control groups (Figure 1). The animals’ body weights were assessed before the first treatment on Day 1 and after the last drug treatment on Day 4. Core body temperatures were assessed 30 min after the first drug treatment on Day 1 using a thermometer equipped with a rectal probe (Physiotemp BAT-12, Physiotemp Instruments Inc., NJ, United States). One week after the final drug treatment, on Day 11, animals were administered manganese dichloride (MnCl_2_) as MEMRI contrast agent through the implantation of manganese-releasing osmotic minipumps. On day 18, following a week of manganese exposure and two weeks after the final drug treatments, animals were anesthetized and subjected to MEMRI brain imaging. Immediately following the completion of the scan, animals were sacrificed (while still under isoflurane anesthesia), and brain tissues were collected and stored for Golgi staining.

### MRI Imaging

#### MnCl_2_ Administration

Manganese chloride was dissolved into Tris-buffered saline (pH 7.4) and administered with osmotic minipumps (Alzet, model 2001) that delivered 200 µL of MnCl_2_ (1 μL/h) during a 7-days infusion, corresponding to a total MnCl_2_ dose of 120 mg/kg (den Hollander et al., 2015). The concentration of MnCl_2_ in the pumps was adjusted according to the body weight of the animals. Prior to implantation, the pumps were primed overnight in saline at 37°C. Animals were anesthetized with isoflurane (Vetflurane, Virbac Animal Health, United Kingdom) and the pumps were implanted subcutaneously on the dorsum, slightly caudal to the scapulae. For post-surgical analgesia, animals received a subcutaneous injection of carprofen (5 mg/kg, Pfizer Animal Health, Belgium) immediately after implantation of the pumps.

#### Magnetic Resonance Imaging Data Acquisition

Animals were transferred from the vivarium to the imaging facility on the day of the MEMRI imaging. MEMRI imaging was performed in a 9.4-T horizontal magnet (Bruker Biospec, Ettlingen, Germany) using a volume coil transmitter/4-channel surface coil receiver pair (Rapid Biomedical, Rimpar, Germany). During the experiments, the animals were anesthetized with isoflurane (5% induction, 1–2% maintenance, 70:30 N_2_:O_2_ gas mixture at 2 L/min) and placed inside a holder with breath monitoring (60–80 breaths per minute) and temperature control (37°C) using warm water. Accurate positioning of the head was ascertained with the help of scout images. T1-weighted images were acquired using a three-dimensional rapid acquisition-relaxation enhanced (RARE) pulse sequence (RARE factor = 6, repetition time = 200 ms, effective echo time = 9 ms, flip angle = 180°, number of averages = 12, field of view = 32 × 19.2 × 14 mm^3^, matrix size = 160/96/70, resulting in 0.2 × 0.2 × 0.2 mm^3^ voxel resolution. The total imaging time was approximately 1 h.

#### Magnetic Resonance Imaging Data Processing

All MRI images were converted to Analyze format, scaled up by a factor of 10 and spatially preprocessed with a custom-developed MATLAB (version R2017) functions using a pipeline described previously (den Hollander et al., 2015). In brief, T1-weighted and brain-extracted images were spatially normalized using a rat brain template co-registered to a rat brain atlas (Schwarz et al., 2006) by a 12-parameter affine transformation using the FSL/FLIRT tool (Jenkinson et al., 2002). This template was co-registered to the digitized Paxinos and Watson atlas (Paxinos and Watson, 2009), which enabled atlas-based generation of region-of-interest (ROI) masks for further detailed anatomical analysis. Spatially normalized were smoothed with a 0.4 × 0.4 × 0.4 mm^3^ full width at half-maximum Gaussian kernel to improve signal-to-noize ratio. For creating statistical parametric maps of differential brain activation between experimental groups, the groups were compared by performing voxel-wise independent two-tailed t-tests using SPM8 (version 6313, www.fil.ion.ucl.ac.uk/spm/).

Despite adjusting the MnCl_2_ concentration for individual rats based on the body weight, systemic administration can lead to inter-individual differences in Mn^2+^ accumulation in the brain, causing brain activation-independent differences in the mean global signal intensity between individuals. Therefore, the mean global intensity was included as a covariate in the general linear model on a voxel-by-voxel basis (Friston et al., 1990). The resulting statistical parametric maps were thresholded voxel-wise using a significance level of *p* < 0.0001 and then cluster-size thresholded with a threshold of k = 32 voxels, resulting in an overall significance level of *p* < 0.01 corrected for multiple comparisons across the whole brain. k was computed based on Monte Carlo simulations.

To describe further activation differences between the groups at anatomically specified brain areas, three-dimensional masks were created with the WFU_PickAtlas tool (Maldjian et al., 2003). The ROI masks were then applied to the SPM contrast files generated previously using the REX tool (Duff et al., 2007). For comparing the groups across selected ROIs, mean ROI intensity values were extracted including all voxels within the ROIs in the analysis.

### Golgi Staining

Brain regions were selected based on the results from voxel-wise and ROI analyses. Particularly, regions with notable deactivation and distinguishable morphological dendritic spine profiles were chosen. The analyzed sections were 2.16 to 1.08 mm for the nucleus accumbens and caudate-putamen and −3.00 mm to −3.24 mm for the dorsal hippocampus. Coordinates correspond to Bregma in Paxinos’ atlas space (Paxinos and Watson, 2009).

#### Sample Preparation

Immediately following the completion of the MRI scan, animals were sacrificed by decapitation and brains were removed and a commercial Golgi staining kit (FD Rapid GolgiStain™ Kit; PK401, FD NeuroTechnologies Inc., Columbia, MD, United States) was used to stain the brain samples. In short, the removed brains were immersed in an impregnation solution. After 14 days of impregnation, the brains were frozen using isopentane and stored in a −80°C freezer.

The brains were then sectioned into 100-μm-thick coronal sections and stained in a multistep staining protocol on gelatin-coated slides (Objektträger 50 K, O. Kindler GmbH; Freiburg, Germany; gelatin powder, Sigma, St. Louis, MO, United States). The regions were imaged in bright-field mode under ×100 oil-immersion objective (EC Plan-Neofluar, NA 1.3) in z-stacks with an AxioImager Z2 microscope and AxioCam 105 camera (Carl Zeiss AG, Oberhocken, Germany). During the imaging process, the correct brain regions were identified, and then z-stacks were taken from each brain region (four z-stacks from both hemispheres), roughly from the same location within the area, keeping the order of the branch of the dendrite (second to third) constant. The imaging was performed blindly, so that the researcher did not know the treatment group of individual sections.

#### Data Analysis

For the Golgi-stained neurons, spine densities and morphologies were analyzed using previously published pipeline for efficient and unbiased classification of dendritic spines (Risher et al., 2014). In short, the previously acquired images were converted to an analyzable file format using ImageJ (version 1.52p; https://imagej.nih.gov/ij/). The converted images were then imported to Reconstructs software (1.1.0.0, https://github.com/SynapseWeb/Reconstruct). All spines within approximately 10 μm length of previously acquired dendrites were manually marked by identifying the base and the top of the spine. The data from the Reconstructs software were then exported to csv-files. Those files were imported to R-studio (2019, version 1.2) and were classified using following criteria: “branched” spines: more than one head; “stubby”: length-width ratio ≤1; “mushroom”: width >0.6 µm; “filopodia”: length >2 μm; “thin”: length-width ratio >1 and “longThin”: length >1 µm. About 860 dendritic segments, approximately 4 segments per animal per hemisphere per brain region, were analyzed with about 30 spines per 10 μm dendrite, resulting in a total of about 25,000 classified spines. The total number per spine type per brain region was divided by the total length of analyzed dendrites per brain region, which resulted in one datapoint per animal per brain region per spine type for the statistical analysis.

### Statistics

Analyses were performed using SPSS (version 24.0.0.1), MATLAB (version R2017), R (version 3.4.4) and GraphPad Prism (version 7). Body temperatures and weights were compared with One-Way analysis of variance (ANOVA) followed by Tukey HSD post-hoc test. The overall effects of treatments on the mean ROI MEMRI signal intensity were assessed using a two-way (brain region × treatment) ANOVA with Greenhouse-Geisser correction. Individual regions were subsequently compared with individual one-way ANOVAs with Fisher’s LSD post-hoc tests. The spine types were compared using individual one-way ANOVAs for each spine type and brain region.

## Results

### Body Temperature and Weight

**FIGURE 2:**
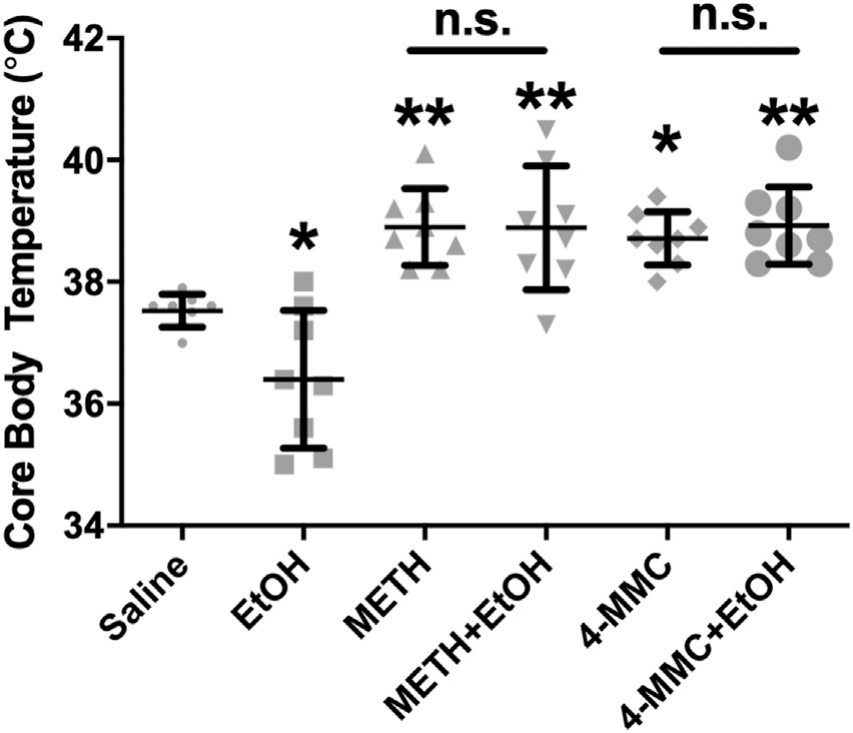
Effect of treatment on body temperature ethanol (EtOH) decreased while all other treatments including the stimulants, increased core body temperatures as assessed 30 min after the first drug administration on Day 1 of the experiment. The data points are means ± SEM for *n* = 8 per group. *p < 0.05 and **p < 0.01 compared with saline-control (Tukey test); n. s., not significant. 4-MMC, 4-methylmethcathinone; EtOH, ethanol; METH, methamphetamine.

A significant effect of drug treatment on core body temperature assessed 30 min after the first drug administration was observed (F_5,47_ = 15.45, *p* < 0.0001). Post-hoc testing revealed a significant decrease in body temperature vs. vehicle only in the group treated with EtOH, whereas a significant increase in body temperature was observed in all other groups that received METH or 4-MMC (Figure 2). Notably, concurrent treatment with EtOH had no modulatory effect on body temperatures in animals treated with the stimulants.

There were no differences in body weights between groups prior to the drug treatments on Day 1 (F _5, 47_ = 0.20, n. s.) or at the end of the drug treatments on Day 4 (F_5,47_ = 1.63, n. s.), indicating that the binge-treatment regimen was well tolerated in all groups.

### Manganese-Enhanced Magnetic Resonance Imaging

#### Voxel-wise

**FIGURE 3:**
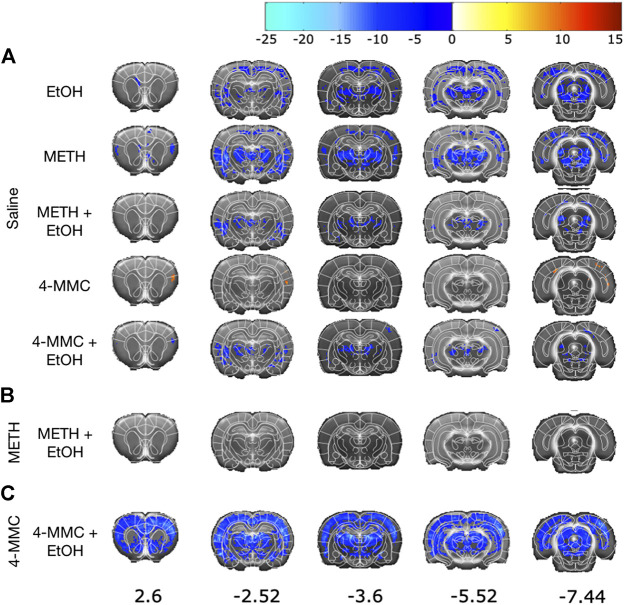
Effect of treatment on brain activity. Statistical color-coded t-maps (thresholded at *pc* < 0.01) are superimposed on T_2_-weighted sections from the brain template (Schwarz et al., 2006), with the corresponding atlas sections (Paxinos and Watson, 2009) manually overlaid. **(A)** All treatments compared to the saline control. **(B)** METH + EtOH treatment compared to METH treatment. **(C)** 4-MMC + EtOH treatment compared to 4-MMC treatment. EtOH, ethanol; METH, methamphetamine; 4-MMC, 4-methylmethcathinone.

All treatments except 4-MMC induced long-term deactivation compared with saline vehicle in multiple cortical regions including the primary motor cortex and secondary somatosensory cortex (Figure 3A). The deactivations were not limited to the cortex but were also present in the midbrain and striatum. Conversely, 4-MMC caused statistically significant activations in the primary and secondary somatosensory cortices and in some regions of the midbrain including the tectum.

The thresholded brain maps of 4-MMC + EtOH compared with 4-MMC alone (Figure 3C) showed deactivations in similar regions as in the METH vs saline analysis. METH + EtOH group looked very similar to groups of METH alone and EtOH alone (Figure 3B).

#### Regions of Interest

Signal intensities from 34 ROIs expressed as intensities relative to the saline-treated control group are shown in Table 1. A two-way ANOVA with treatment as between-subjects factor and brain region as within-subjects factor revealed a significant main effect of treatment (F_5,42_) = 3.42, *p* < 0.05), as well as a significant Greenhouse-Geisser–corrected main effect of region (F _3.50, 1386_ = 13.89, *p* < 0.0005) and treatment × region interaction (F_17.48, 1386_ = 2.07, *p* < 0.05).

**TABLE 1:** Effects of METH and 4-MMC alone or in combination with EtOH on magnetic resonance imaging signal intensity in pre-defined ROIs, two weeks following the last dose of the binge treatment regimen. The mean intensity values in each ROI were normalized (saline = 1). All data are expressed as mean ± SEM. *N* = 8 per group. Significant *p* values (*p* < 0.05) are marked by bolded text. Only significant *p* values with ANOVA main effect are bolded. EtOH, alcohol; METH, methamphetamine; 4-MMC, 4-methylmethcathinone; ROI, region of interest; SEM, standard error of the mean.

ROI	MRI intensity value (mean ± SEM)						ANOVA		Post-hoc *p*-value						
	Saline	EtOH	METH	METH + EtOH	4-MMC	4-MMC + EtOH	F	*p*	Saline vs EtOH	Saline vs METH	Saline vs METH + EtOH	METH vs METH + EtOH	Saline vs 4-MMC	Saline vs 4-MMC + EtOH	4-MMC vs 4-MMC + EtOH
**Dopamine system**															
Nucleus accumbens core	1.00	0.89	0.89	0.89	1.05	0.91	3.57	**0.009**	**0.037**	**0.040**	**0.046**	0.949	0.351	0.086	**0.010**
	±0.04	±0.05	±0.03	±0.03	±0.03	±0.03									
Nucleus accumbens shell	1.00	0.89	0.89	0.90	1.04	0.91	3.11	**0.018**	**0.042**	**0.036**	0.068	0.770	0.440	0.101	**0.018**
	±0.04	±0.05	±0.03	±0.03	±0.04	±0.03									
Caudate/putamen (striatum)	1.00	0.89	0.89	0.90	1.05	0.92	3.77	**0.007**	**0.037**	**0.032**	**0.045**	0.877	0.298	0.103	**0.009**
	±0.04	±0.04	±0.02	±0.04	±0.03	±0.03									
Lateral globus pallidus	1.00	0.87	0.88	0.89	1.04	0.90	3.19	**0.016**	**0.025**	**0.036**	**0.047**	0.729	0.523	0.093	**0.023**
	±0.05	±0.05	±0.03	±0.03	±0.04	±0.03									
Substantia nigra	1.00	0.99	0.86	0.93	0.97	0.87	1.43	0.232	0.888	0.055	0.344	0.654	0.683	0.080	0.166
	±0.05	±0.05	±0.03	±0.05	±0.04	±0.07									
Ventral tegmental area	1.00	0.95	0.90	0.94	1.05	0.92	1.98	0.101	0.431	0.075	0.279	0.488	0.383	0.160	0.028
	±0.05	±0.04	±0.02	±0.04	±0.04	±0.05									
**Cerebral cortex**															
Insular cortex	1.00	0.93	0.89	0.92	1.07	0.93	2.65	**0.036**	0.201	0.055	0.185	0.536	0.242	0.256	**0.024**
	±0.05	±0.04	±0.03	±0.05	±0.04	±0.04									
Primary auditory cortex	1.00	0.90	0.89	0.91	1.09	0.94	3.23	**0.015**	0.096	0.076	0.150	0.726	0.153	0.293	**0.016**
	±0.06	±0.03	±0.02	±0.05	±0.04	±0.05									
Secondary auditory cortex	1.00	0.90	0.88	0.91	1.08	0.93	3.23	**0.015**	0.104	0.059	0.141	0.663	0.172	0.253	**0.015**
	±0.05	±0.03	±0.02	±0.05	±0.04	±0.05									
Cingulate cortex	1.00	0.89	0.89	0.90	1.07	0.91	4.34	**0.003**	**0.040**	**0.037**	0.051	0.884	0.166	0.094	**0.003**
	±0.04	±0.04	±0.03	±0.03	±0.03	±0.03									
Ectorhinal cortex	1.00	0.93	0.87	0.96	1.06	0.92	1.51	0.208	0.342	0.103	0.567	0.283	0.433	0.295	0.071
	±0.06	±0.04	±0.03	±0.06	±0.06	±0.06									
Frontal association cortex	1.00	0.90	0.90	0.90	1.07	0.90	3.99	**0.005**	0.056	0.061	0.062	0.993	0.171	0.081	**0.003**
	±0.04	±0.05	±0.03	±0.04	±0.03	±0.03									
Infralimbic cortex	1.00	0.90	0.89	0.92	1.06	0.93	3.08	**0.019**	0.055	**0.046**	0.125	0.499	0.304	0.173	**0.020**
	±0.05	±0.04	±0.03	±0.03	±0.04	±0.03									
Primary motor cortex	1.00	0.89	0.89	0.89	1.06	0.92	4.09	**0.004**	**0.042**	**0.034**	**0.035**	0.904	0.224	0.128	**0.008**
	±0.04	±0.05	±0.03	±0.04	±0.03	±0.03									
Secondary motor cortex	1.00	0.90	0.90	0.91	1.08	0.93	3.83	**0.006**	0.063	0.063	0.088	0.874	0.138	0.190	**0.007**
	±0.04	±0.05	±0.03	±0.04	±0.03	±0.03									
Orbital cortex	1.00	0.90	0.90	0.92	1.07	0.92	3.36	**0.012**	0.076	0.068	0.128	0.993	0.184	0.154	**0.008**
	±0.04	±0.05	±0.03	±0.03	±0.03	±0.03									
Perirhinal cortex	1.00	0.96	0.92	0.96	1.12	0.97	1.85	0.124	0.583	0.259	0.566	0.753	0.107	0.719	0.051
	±0.07	±0.04	±0.02	±0.06	±0.05	±0.06									
Prelimbic cortex	1.00	0.90	0.90	0.90	1.06	0.91	4.05	**0.004**	**0.043**	**0.043**	0.050	0.575	0.212	0.076	**0.004**
	±0.04	±0.05	±0.03	±0.03	±0.03	±0.03									
Parietal association cortex	1.00	0.90	0.89	0.90	1.08	0.92	4.50	**0.002**	0.050	**0.031**	**0.042**	0.943	0.140	0.140	**0.004**
	±0.04	±0.04	±0.02	±0.04	±0.03	±0.03									
Retrosplenial cortex	1.00	0.94	0.95	0.95	1.12	0.97	3.83	**0.006**	0.218	0.285	0.295	0.448	**0.021**	0.556	**0.004**
	±0.04	±0.03	±0.02	±0.04	±0.03	±0.04									
Primary somatosensory cortex	1.00	0.90	0.93	0.94	1.12	0.96	4.61	**0.002**	0.054	0.200	0.297	0.545	**0.024**	0.491	**0.003**
	±0.04	±0.04	±0.03	±0.04	±0.04	±0.03									
Secondary somatosensory cortex	1.00	0.91	0.87	0.90	1.05	0.91	3.33	**0.013**	0.109	**0.019**	0.066	0.797	0.373	0.086	**0.012**
	±0.05	±0.04	±0.02	±0.04	±0.04	±0.04									
**Subcortical**															
Amygdala	1.00	0.90	0.87	0.94	1.08	0.88	3.17	**0.016**	0.116	0.052	0.354	0.295	0.219	0.078	**0.004**
	±0.05	±0.04	±0.04	±0.04	±0.04	±0.05									
Bed nucleus of the stria terminalis	1.00	0.88	0.89	0.89	1.04	0.90	3.15	**0.017**	**0.036**	**0.039**	0.054	0.878	0.478	0.079	**0.016**
	±0.04	±0.05	±0.03	±0.03	±0.04	±0.03									
*Hippocampus*, dorsal	1.00	0.91	0.96	0.97	1.15	0.99	4.84	**0.001**	0.107	0.407	0.520	0.844	**0.006**	0.805	**0.002**
	±0.05	±0.04	±0.02	±0.04	±0.04	±0.04									
*Hippocampus*, ventral	1.00	0.92	0.77	0.84	0.96	0.78	4.53	**0.002**	0.199	**0.001**	**0.017**	0.262	0.578	**0.001**	**0.006**
	±0.05	±0.05	±0.03	±0.04	±0.04	±0.06									
Thalamus, dorsal	1.00	0.91	0.88	0.89	1.04	0.91	3.78	**0.006**	0.058	**0.014**	**0.025**	0.830	0.447	0.056	**0.011**
	±0.04	±0.04	±0.02	±0.03	±0.03	±0.03									
Thalamus, ventral	1.00	0.90	0.97	0.99	1.15	1.01	4.84	**0.001**	0.071	0.624	0.825	0.788	**0.006**	0.889	**0.008**
	±0.04	±0.03	±0.03	±0.04	±0.04	±0.04									
Subthalamic nucleus	1.00	0.93	0.94	0.99	1.13	0.97	2.85	**0.026**	0.257	0.358	0.811	0.304	**0.036**	0.585	**0.009**
	±0.05	±0.04	±0.03	±0.05	±0.04	±0.04									
Hypothalamus	1.00	0.97	0.93	0.98	1.06	0.96	0.88	0.505	0.673	0.307	0.726	0.993	0.363	0.569	0.143
	±0.06	±0.03	±0.03	±0.05	±0.04	±0.04									
Interstitial nucleus of the posterior limb of the anterior commissure	1.00	0.89	0.88	0.90	1.05	0.91	3.40	**0.011**	**0.046**	**0.026**	0.058	0.627	0.396	0.086	**0.012**
	±0.04	±0.04	±0.03	±0.04	±0.04	±0.03									
Raphe nucleus	1.00	0.93	0.88	0.92	1.05	0.92	3.02	**0.020**	0.207	**0.021**	0.110	0.897	0.353	0.116	**0.014**
	±0.04	±0.03	±0.04	±0.04	±0.04	±0.04									
Superior colliculus	1.00	0.93	0.92	0.93	1.10	0.95	4.43	**0.003**	0.132	0.097	0.144	0.594	**0.036**	0.327	**0.003**
	±0.04	±0.03	±0.02	±0.04	±0.04	±0.03									
Substantia innominata	1.00	0.90	0.84	0.86	0.98	0.86	3.53	**0.009**	0.061	**0.003**	**0.009**	0.839	0.704	**0.011**	**0.026**
	±0.04	±0.04	±0.03	±0.03	±0.04	±0.04									

Only significant p values with ANOVA main effect are bolded.

Confirming previous results (den Hollander et al., 2015), METH- and 4-MMC-treated groups induced very different signal intensities throughout the brain (Table 1), with METH-treatment reducing the signal and 4-MMC increasing it in several brain regions as compared to the saline-treated group. METH produced significant decreases in brain activity in DA terminal regions (Table 1) as well a multitude of cortical and subcortical regions, including the cingulate and limbic cortex, ventral hippocampus, dorsal thalamus and raphe nucleus. 4-MMC treatment resulted in increased brain activity, although this effect did not reach statistical significance in any DA terminal region and was limited to the retrosplenial cortex and primary somatosensory cortex, as well as the dorsal hippocampus, thalamic areas and the superior colliculus.

In EtOH-treated animals, a decrease in brain activity was observed as compared with saline-treated group (Table 1). This effect was most notable in DA terminal regions, such as the nucleus accumbens and caudate-putamen and throughout several cortical and subcortical regions, including the cingulate and prelimbic cortex and the bed nucleus of the stria terminalis. Addition of EtOH to the METH treatment yielded similar deactivations in signal intensity vs. saline as EtOH or METH alone. Interestingly, the activation seen in the 4-MMC group was not observed in the group treated with 4-MMC + EtOH. Conversely, in the 4-MMC + EtOH treated group the deactivation pattern strongly resembled the deactivation pattern seen in the group treated with METH.

### Golgi Staining

**FIGURE 4:**
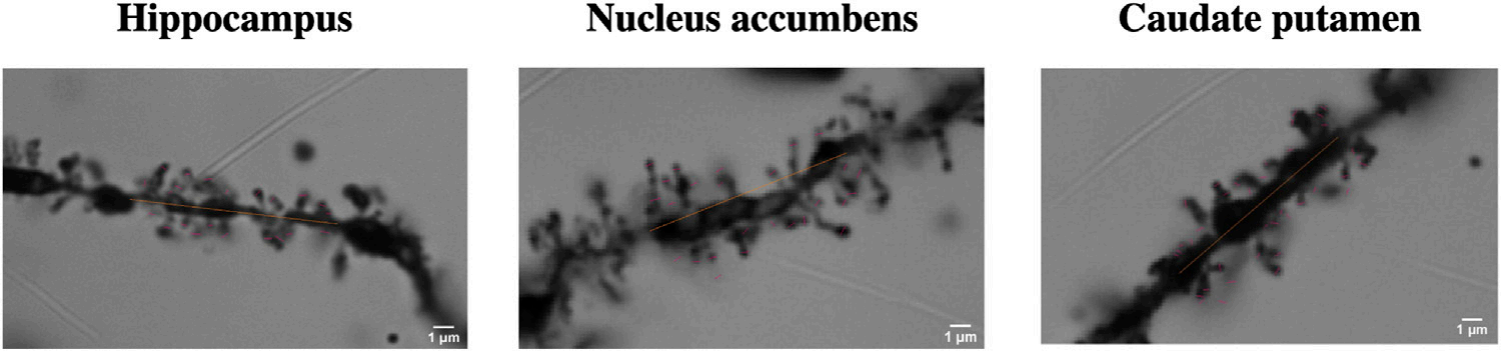
One representative dendrite section from the hippocampus, nucleus accumbens and caudate putamen shown as an example of the labeling process.

Brain regions which showed the most notable changes and had distinguishable dendritic spine morphology were chosen for analysis. With those criteria hippocampal CA1, nucleus accumbens and caudate-putamen were selected for analysis (for representative examples, please see Figure 4).

**FIGURE 5:**
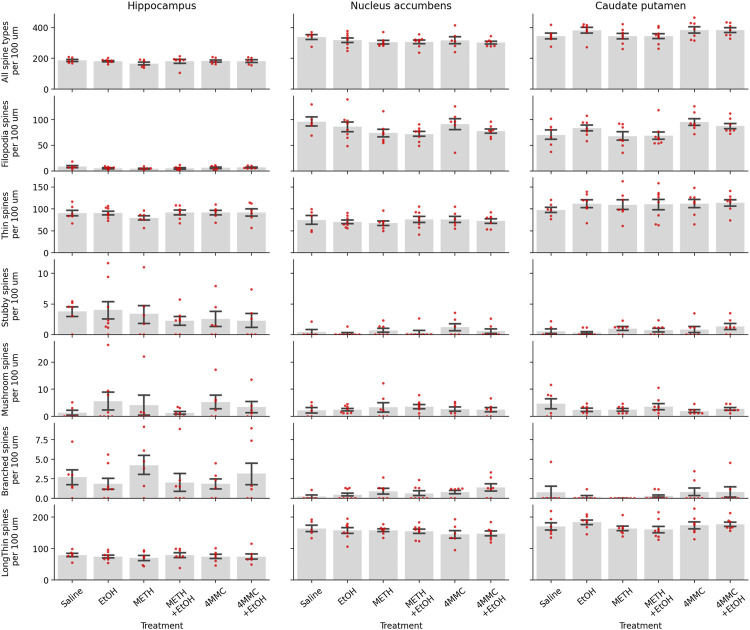
Spine density per spine type shown in the hippocampus, nucleus accumbens and caudate putamen. Individual data points shown in red, bars represent data as mean ± SEM.

The hippocampal CA1 region was analyzed for spine elements using a total of 39 brains (saline-control *n* = 6, METH *n* = 6, 4-MMC *n* = 6, EtOH *n* = 8, METH + EtOH *n* = 7, and 4-MMC + EtOH *n* = 6). No significant differences between the treatment groups were seen in the total number of spines (F _5, 33_ = 0.59, n. s.), filopodia-type spines (F_5, 33_ = 1.00, n. s.), thin-type spines (F_5, 33_ = 0.57, n. s.), stubby-type spines (F_5, 33_ = 0.40, n. s.), mushroom-type spines (F_5,33_ = 0.57, n. s.), branched-type spines (F_5, 33_ = 0.69, n. s.) and long-thin-type spines (F_5, 33_ = 0.20, n. s.) (Figure 5).

Nucleus accumbens dendrites were analyzed for spine elements using a total of 41 brains (saline-control *n* = 5, METH *n* = 7, 4-MMC *n* = 6, EtOH *n* = 8, METH + EtOH *n* = 8, 4-MMC + EtOH *n* = 7). No significant differences between treatment groups were seen in medium spiny neurons, total number of spines (F_5, 35_ = 0.63, n. s.), filopodia-type spines (F_5, 35_ = 1.29, n. s.), thin-type spines (F_5, 35_ = 0.25, n. s.), stubby-type spines (F_5, 35_ = 0.93, n. s.), mushroom-type spines (F_5, 35_ = 0.28, n. s.), branched-type spines (F_5, 35_ = 1.37, n. s.) and long-thin-type spines (F_5, 35_ = 0.51, n. s.) (Figure 5).

The dorsomedial caudate-putamen was analyzed for spine elements using a total of 42 brains (saline-control *n* = 6, METH *n* = 7, 4-MMC *n* = 7, EtOH *n* = 7, METH + EtOH *n* = 8, and 4-MMC + EtOH *n* = 7). No significant differences between treatment groups were seen in the total number of spines (F_5, 36_ = 1.27, n. s.), filopodia-type spines (F_5, 36_ = 2.45, n. s.), thin-type spines (F_5, 36_ = 0.28, n. s.), stubby-type spines (F_5, 36_ = 0.87, n. s.), mushroom-type spines (F_5, 36_ = 0.98, n. s.), branched-type spines (F_5, 36_ = 0.67, n. s.) and long-thin-type spines (F_5, 36_ = 0.77, n. s.) (Figure 5).

## Discussion

Here we show that co-administration of EtOH with 4-MMC in a binge-abuse model produces a widespread pattern of neuronal deactivation in monoamine-rich brain areas when assessed with MEMRI two weeks after the last drug administration. The 4-MMC + EtOH deactivation pattern was very similar to the one observed following METH administration. This pattern of widespread deactivation is in contrast with the effect seen when 4-MMC was administered alone. 4-MMC increased, rather than decreased, neuronal activity in an anatomically more limited manner, and occurring in areas not strongly innervated by DA axons. These findings regarding 4-MMC alone were in line with our previous results, indicating good reproducibility of the MEMRI method (den Hollander et al., 2015). However, when examining neuronal morphology in the hippocampus, nucleus accumbens and caudate-putamen, the three regions showing the strongest neuronal deactivation in animals treated with 4-MMC and EtOH, we found no morphological changes to dendrites suggestive of long-term neurotoxic effects.

When administered alone, 4-MMC produces a subtle pattern of long-term neuronal activation in regions like the retrosplenial and primary somatosensory cortex, as well as the dorsal hippocampus, thalamic areas and the superior colliculus. These areas are not strongly innervated by DA, and the observed changes plausibly do not reflect DA neurotoxicity. A potential explanation for the increased neuronal activity is that during 4-MMC intoxication, it has been shown that plasma cortisol levels are elevated (Papaseit et al., 2020). Elevation of cortisol levels in acute stress via the serotonergic effects on the hypothalamo-pituitary-adrenal axis has been shown to activate regions important for memory consolidation, like the hippocampus (Herman and Cullinan, 1997; Kim et al., 2015). Interesting in this regard is that 4-MMC was shown to reduce memory performance 2 weeks after a similar binge-regimen as employed in this study without causing any reductions in monoamine levels that could be indicative of toxicity (den Hollander et al., 2013).

The pattern of widespread neuronal deactivation seen following treatment with 4-MMC in combination with EtOH as well as following METH, either alone or in combination with EtOH, is striking considering the anatomical correlation of the deactivation pattern with brain regions innervated by DA nerve ending and typically implicated in neurotoxicity of amphetamines (Ares-Santos et al., 2014). Nonetheless, even the areas showing the strongest deactivation did not display signs of neurotoxicity as assessed by subsequent Golgi staining and dendritic spine analysis. This raises the question what the exact neurochemical correlates of the MEMRI deactivation pattern are. Co-administration of 4-MMC with EtOH lowers synaptic transmitter function markers for DA and 5-HT, while elevating oxidative stress markers at least in high ambient temperatures (Ciudad-Roberts et al., 2016). It is thus possible that the 4-MMC and EtOH-induced deactivation observed here reflects the long-term functional effects of increased oxidative stress-related adaptation in synaptic terminals. However, it should be noted that deactivation in brain regions largely overlapping with those in the 4-MMC/EtOH-treated group were observed in the group treated with EtOH only. Considering that the EtOH dose employed in this study was moderate it is worth noting that neuronal deactivation as assessed by MEMRI should not be considered as conclusive evidence of neurotoxicity, although such a correlation has been observed previously (Weng et al., 2016). Similarly, neurotoxicity may have occurred in the 4-MMC group in a way that is not reflected in the MEMRI data. The exact neurochemical correlates of altered activation patterns remain to be determined and until then the data must be interpreted with relative caution.

Despite these caveats, our results appear in agreement with previous studies that have generally failed to observe neurotoxicity following administration of 4-MMC at normal ambient temperatures (Angoa-Perez et al., 2012; Baumann et al., 2012; Angoa-Perez et al., 2013; Angoa-Perez et al., 2014; den Hollander et al., 2015; Ciudad-Roberts et al., 2016). Although there are some reports of neurotoxicity as assessed by levels of monoamines, their transporters and tyrosine hydroxylase (Martinez-Clemente et al., 2014; Kaminska et al., 2018), 4-MMC neurotoxicity is primarily observed when administered under exacerbating conditions, specifically increased ambient temperatures (Hadlock et al., 2011; Lopez-Arnau et al., 2015; Pantano et al., 2017). This raises the question as to what pharmacological differences between 4-MMC and METH result in the latter much more readily inducing neurotoxicity in animal models. Anneken et al. (2018) hypothesized that the proposed difference could be due to differences in how METH and 4-MMC access the drug-releasable pool of DA. However, their experiments showed that combining the drugs with l-DOPA, which increases the size of the release pool, did in fact not precipitate any 4-MMC neurotoxicity while enhancing the toxic effects of METH. In a subsequent experiment, the same group investigated whether differences in how the two drugs modulate core body temperatures may be responsible for differences in toxicity using tryptophan hydroxylase 2 knockout mice lacking brain serotonin. The rationale for this experiment was the observation that while both METH and 4-MMC produce hyperthermia, 4-MMC has also been reported to produce a brief hypothermic response. However, they reported that although the knockout mice did not experience the characteristics hypothermic response, no increase in toxicity was observed in the knockouts vs. the wild-types (Anneken et al., 2019). Another possibility is that 4-MMC has a different effect of mitochondria than METH, which has been associated with mitochondrial-dependent mechanisms of toxicity (Shin et al., 2018). However, 4-MMC in fact appears to also affect mitochondria. It has been shown to impair mitochondrial complexes II and IV, collapse mitochondrial membrane potential, induce mitochondrial swelling and lower mitochondrial respiration *in vitro* (den Hollander et al., 2014; Naserzadeh et al., 2019). Nonetheless, in the case of 4-MMC, these *in vitro* results do not generally appear to translate into measurable neurotoxicity *in vivo*. Thus, the exact mechanism(s) underlying the observed differences in toxicity between 4-MMC and METH remain to be elucidated.

In this study, core body temperatures vs. the control group were lowered after EtOH but increased after 4-MMC and METH, in line with results from previous studies (Lomax et al., 1980; Myles and Sabol, 2008; den Hollander et al., 2015). Notably, co-administration of EtOH with 4-MMC and METH neither exacerbated nor blocked the hypothermic effects of these drugs. Since hyperthermia was seen in stimulant-treated groups and not affected by EtOH co-administration, any differences observed between groups were not due to a differing hyperthermic reaction in this study.

Golgi staining followed by spine analysis has been suggested to be an effective and unbiased way to assess neuronal changes at the dendritic spine level (Risher et al., 2014). This has also proven to be the case with stimulant-induced changes in dendritic spines (Robinson and Kolb, 1997; Robinson and Kolb, 1999). However, in the present experiment, no statistically significant alterations were found in the spine analysis in three relevant brain regions. This lack of changes could be due to the limitations in Golgi staining, such as variability of staining intensity, variability in localizing the brain area within thick brain sections or the random nature at which neurons of different types are stained which is inherent to the Golgi method. Stimulant-induced increases in striatal spines have been found in studies in which prolonged psychomotor sensitization has been detected (Li et al., 2004), but our slightly heavier regimen was aimed at finding possible neurotoxicity without sensitization (den Hollander et al., 2015). Another aspect to be taken into account is the fact that changes in spine formation, deformation and maturation can be very fast and bidirectional (Nagerl et al., 2004; Berry and Nedivi, 2017). To our knowledge there has been no previous research to assess the temporal stability of stimulant-induced alterations in spine morphology. It is possible that stimulant-induced alterations in spine morphology are not stable enough to persist following the 2-weeks recovery period after treatments. Furthermore, Golgi-staining stains post-synaptic parts of the synapse whereas the stimulant-induced neurotoxicity has previously been associated with the axonal part of the synapse but at higher doses, from 20 mg/kg and up (Zhu et al., 2006) than used in this experiment. And therefore, our dose of 5 mg/kg METH might not have been sufficient to cause axonal apoptosis which would then cause dendritic spine deformation. Lastly, it is conceivable that the isoflurane anesthesia employed during the MEMRI scanning could have produced alterations or reversals in morphology considering the fact that isoflurane has previously been associated with activation of neurotrophic signaling, but not with spine structure changes (Antila et al., 2017).

A limitation of this study is that only Golgi staining was employed to interpret the neuronal activation patterns observed in MEMRI experiment. The assessments of levels of monoamines or their transporters, as well as other assessments of inflammation and oxidative stress during and following the binge regimen could have provided further insights into potential causative factors. Also, other type of silver staining or terminal deoxynucleotidyl transferase dUTP nick end labeling could be used to assess cell-level neurotoxicity. Moreover, assessments of neurocognitive and neuropsychiatric function such as memory, mood and anxiety tests, could have provided further information about functional effects. These are aspects which will need to be addressed in future studies.

When administered on its own, the effects of 4-MMC on neural activity are subtle, anatomically limited, and likely not reflective of long-term neurochemical perturbations. However, when administered in combination with EtOH, it produces a pattern of widespread neuronal deactivation in brain regions densely innervated by DA neurons similar to what is seen after METH. The deactivated regions nonetheless do not show clear morphological changes indicative of toxicity. This suggests the deactivation pattern is likely due to long-term neurochemical perturbations that are substantial but not so severe as to produce overt neurotoxicity.

In conclusion, this study suggests that binge-treatment with 4-MMC on its own does not substantially alter brain activity, but in combination with EtOH may result in long-term reductions in brain activity. This is important from a harm-reduction perspective, although further studies are needed to outline the exact neurochemical changes responsible for the altered activity patterns.

## Data Availability

The raw data supporting the conclusions of this article will be made available by the authors, without undue reservation.
